# Predictive Scale for Amyloid PET Positivity Based on Clinical and MRI Variables in Patients with Amnestic Mild Cognitive Impairment

**DOI:** 10.3390/jcm11123433

**Published:** 2022-06-15

**Authors:** Min Young Chun, Geon Ha Kim, Hee Kyung Park, Dong Won Yang, SangYun Kim, Seong Hye Choi, Jee Hyang Jeong

**Affiliations:** 1 Department of Neurology, Samsung Medical Center, Sungkyunkwan University School of Medicine, Seoul 06351, Korea; myc88@hanmail.net; 2 Department of Neurology, Ewha Womans University Mokdong Hospital, Ewha Womans University College of Medicine, Seoul 07985, Korea; geonha@ewha.ac.kr (G.H.K.); jenhkpark@gmail.com (H.K.P.); 3 Division of Psychiatry, Department of Mental Health Care of Older People, University College London, London W1T 7NF, UK; 4 Department of Neurology, St. Mary’s Hospital, College of Medicine, The Catholic University of Korea, Seoul 06591, Korea; neuroman@catholic.ac.kr; 5 Department of Neurology, Seoul National University College of Medicine & Seoul National University Bundang Hospital, Seongnam 13620, Korea; neuroksy@snu.ac.kr; 6 Department of Neurology, Inha University School of Medicine, Inchon 22232, Korea; 7 Department of Neurology, Ewha Womans University Seoul Hospital, Ewha Womans University College of Medicine, Seoul 07804, Korea

**Keywords:** mild cognitive impairment, Alzheimer’s disease, positron emission tomography, apolipoprotein E, medial temporal lobe atrophy

## Abstract

The presence of amyloid-β (Aβ) deposition is considered important in patients with amnestic mild cognitive impairment (aMCI), since they can progress to Alzheimer’s disease dementia. Amyloid positron emission tomography (PET) has been used for detecting Aβ deposition, but its high cost is a significant barrier for clinical usage. Therefore, we aimed to develop a new predictive scale for amyloid PET positivity using easily accessible tools. Overall, 161 aMCI patients were recruited from six memory clinics and underwent neuropsychological tests, brain magnetic resonance imaging (MRI), apolipoprotein E (*APOE*) genotype testing, and amyloid PET. Among the potential predictors, verbal and visual memory tests, medial temporal lobe atrophy, *APOE* genotype, and age showed significant differences between the Aβ-positive and Aβ-negative groups and were combined to make a model for predicting amyloid PET positivity with the area under the curve (AUC) of 0.856. Based on the best model, we developed the new predictive scale comprising integers, which had an optimal cutoff score ≥ 3. The new predictive scale was validated in another cohort of 98 participants and showed a good performance with AUC of 0.835. This new predictive scale with accessible variables may be useful for predicting Aβ positivity in aMCI patients in clinical practice.

## 1. Introduction

Mild cognitive impairment (MCI) has been considered as a transitional state between normal aging and dementia [1]. Since the growth of the elderly population has led to an increase in the prevalence and socioeconomic burden of dementia patients [2], early intervention at the MCI stage and prevention of progression to dementia have become important. In particular, amnestic MCI (aMCI) has a high probability of progression to dementia due to Alzheimer’s disease (AD) [3,4].

AD is neuropathologically diagnosed based on the deposition of amyloid-β (Aβ) plaques and pathologic tau proteins [5]. As the pathologic changes occur in the brain before the symptoms of AD appear [6], modalities for examining the presence of AD pathology, such as cerebrospinal fluid (CSF) analysis and amyloid positron emission tomography (PET) scans, have been developed to detect AD in the preclinical or prodromal stages. The CSF assay has the advantages of being able to measure not only Aβ but also tau protein [7]. On the other hand, amyloid PET scans are noninvasive and have high reliability in longitudinal studies and center-to-center examinations [7].

Although these AD confirmatory diagnostic modalities have been used as enrollment criteria for research, their application in primary care clinical settings is still difficult. CSF testing is an invasive test that the patients may be reluctant to undergo. Amyloid PET scans have the issue of high cost, availability of PET scanners, and radiation exposure [8,9]. Thus, their widespread use is limited to the first-line approach in clinical trials.

Although several longitudinal studies have developed models for predicting the conversion of MCI to AD dementia, AD was estimated clinically, not pathologically [10,11,12,13]. In addition, a few Aβ-positive (Aβ+) AD prediction models that could replace the confirmatory tests have been suggested using cortical thickness and brain volumetry [14,15,16], or using several blood-based markers, including prostatic acid phosphatase, transthyretin, matrix metalloproteinase 10 [17], and plasma Aβ42/Aβ40 [18]. However, these models used variables that are still difficult to apply in clinical practice. Therefore, it will be useful to develop a predictive scale of Aβ positivity using commonly available variables in aMCI patients.

This study aimed to develop a predictive scale to distinguish amyloid PET positivity among aMCI patients using easily accessible variables for routine clinical practice. These variables included demographic and clinical characteristics of aMCI patients such as age, sex, education, detailed neuropsychological tests including verbal and visual memory tests [11,19,20,21,22], medial temporal lobe atrophy (MTA) [23,24,25,26], and apolipoprotein E (*APOE*) epsilon 4 (ε4) allele [27,28].

## 2. Materials and Methods

### 2.1. Participants

A total of 161 participants with aMCI were recruited from six different memory clinics in South Korea.

Diagnosis of aMCI was based on the Petersen’s criteria for MCI [3]: (1) subjective memory complaint by the subject or an informant; (2) preserved general cognitive function; (3) objective memory impairment below −1.0 standard deviation (SD) of age, sex, and education matched norms on either verbal or visual delayed recall tests; and (4) independent performance of daily life activities. The participants had a global Clinical Dementia Rating (CDR) score of 0.5. All participants underwent standard assessments including clinical history, neurological examination, detailed neuropsychological tests, brain magnetic resonance imaging (MRI), amyloid PET, *APOE* genotype, and laboratory tests. Patients who had a critical illness, stroke, brain tumors, psychiatric diseases, and head trauma with loss of consciousness were excluded from the study population.

The participants were categorized into two groups based on whether the amyloid PET results were Aβ+ or Aβ-negative (Aβ−). For the *APOE* genotype, the participants were classified as carriers or noncarriers of the *APOE* ε4 allele.

The present study was approved by the Institutional Review Board for Human Research of the institution in each center and written informed consent was obtained from all participants (ClinicalTrials.gov identifier: NCT02656498).

### 2.2. Neuropsychological Tests

All participants underwent the Korean-Mini Mental Status Examination (K-MMSE), global CDR, Sum of Boxes of CDR (CDR-SB), and Seoul Neuropsychological Screening Battery (SNSB) [29]. SNSB, a detailed neuropsychological test, is subdivided into the following domains. Memory function domain included Seoul Verbal Learning Test-delayed recall (SVLT-DR) and Rey Complex Figure Test-delayed recall (RCFT-DR). Attention domain included Digit Span Test-forward (DST-forward) and language function domain included Korean-Boston Naming Test (K-BNT). Visuospatial function domain was composed of the RCFT-copy. Frontal/executive function domain was evaluated by Controlled Oral Word Association Test-phonemic (COWAT-phonemic), Korean-Color Word Stroop Test-color reading (K-CWST-CR), Digit Symbol Coding (DSC), and Korean-Trail Making Test-part B (K-TMT-B).

All SNSB variables were converted to z-scores standardized for age, sex, and years of education. Each SNSB variable was considered abnormal when the z-score was lower than −1.0 SD (16th percentiles) of the age, sex, and education-standardized norms. Subjects were classified as aMCI, if either SVLT-DR or RCFT-DR was less than −1.0 SD with independent activity of daily living.

### 2.3. Axial Visual Rating Scale of Medial Temporal Lobe Atrophy

All participants were evaluated with the axial visual rating scale (aVRS) of MTA according to the method developed by Kim et al. [30] as the inter-rater and intra-rater reliability of aVRS were higher than coronal VRS proposed by Scheltens et al. [31] The MTA of each subject was rated twice by two trained neurologists, who were unaware of the clinical information of the participants. The left and right sides of the MTA were evaluated separately, and the larger value was chosen if the left and right values were different [30,32,33]. According to previous studies which found that an MTA score of 2 or higher was associated with progression from aMCI to dementia [24,33,34], MTA variables were classified as presence or absence of MTA on the basis of aVRS of 2. The kappa value for intra-rater reliability of MTA aVRS was 0.820 (95% confidence interval (CI) 0.779–0.862) and inter-rater reliability was 0.752 (95% CI 0.651–0.845).

### 2.4. MRI and PET Imaging Acquisition

MRI scans were obtained using 1.5-Tesla (T) MRI (Siemens Magnetom Avanto, Siemens, Erlangen, Germany), 3.0-T MRI (Philips Achieva, Philips, Best, The Netherlands), 3.0-T MRI (Siemens Magnetom VIDA, Siemens, Erlangen, Germany), 3.0-T MRI (GE Signa Architect, Waukesha, WI, USA), 3.0-T MRI (Philips Ingenia, Philips, Best, The Netherlands), or 3.0-T MRI (Verio, Siemens with a Siemens matrix coil, Erlangen, Germany) machines at six different memory clinics. All MR images included T1-weighted axial, T2-weighted axial, and fluid attenuated inversion recovery (FLAIR) axial images. For axial MR images, the images were taken parallel to the line from the anterior commissure to the posterior commissure.

All participants underwent one of the following two amyloid PET tests: ^11^C-Pittsburgh compound B ([^11^C] PiB) or ^18^F-florbetaben ([^18^F] FBB). Nine subjects underwent [^11^C] PiB PET (Siemens) and 138 subjects underwent [^18^F] FBB PET (Biograph mCT, Siemens) scans. The standard uptake value ratio (SUVR) was obtained for [^11^C] PiB images to determine amyloid PET positivity [35]. [^18^F] FBB PET was evaluated visually by a trained nuclear medicine specialist in each center using brain amyloid plaque load (BAPL) score.

### 2.5. Validation of the Predictive Scale

For the validation cohort, 98 aMCI subjects were recruited from the Inha University hospital in South Korea. The inclusion criteria of the validation cohort were the same as those of the original group. To validate the new predictive scale of amyloid PET positivity, the scale was applied to the validation cohort set. Receiver operating characteristic (ROC) curve analysis was performed to evaluate the predictive performance. Area under the curve (AUC) value, sensitivity, and specificity were calculated at the optimal cutoff score of the newly developed scale.

### 2.6. Statistical Analyses

In this cross-sectional study, Student’s t-test was used to analyze the continuous variables, while the chi-square test was used for the dichotomous variables. Based on the results of the comparison between Aβ+ and Aβ− subjects, potential predictors were selected. Pearson’s correlation analysis was conducted to determine the association between the potential predictors as well as to exclude the correlates.

The selected significant continuous variables were converted to categorical variables to maximize their utility in clinical practice. Since the two groups of Aβ+ and Aβ− subjects were divided based on the average age of 70 years, 1 point was given to those aged 70 or older and 0 point to those under the age of 70. For SNSB, subjects with higher than −1.0 SD were scored as 0, while subjects with lower than −1.0 SD were scored as 1. The presence of MTA was determined and scored as 1 if MTA aVRS was ≥2. If the subject was an *APOE*-ε4 carrier, a score of 1 was given, while if the subject was an *APOE*-ε4 noncarrier, a score of 0 was given.

Univariate logistic regression analysis was applied to predict amyloid PET positivity with each potential predictor. Subsequently, these predictors were combined to develop several models using multivariate logistic regression analysis. The ROC curves for all models were analyzed to assess their predictive ability for amyloid PET positivity. The model with the highest AUC value was selected as the most appropriate model for predicting amyloid PET positivity. To detect multicollinearity between the predictors in the model, variance inflation factor (VIF) statistics were calculated. The calibration of each prediction model was assessed using the Hosmer–Lemeshow goodness-of-fit test.

The most appropriate model could generate a new predictive scale based on the regression β coefficients from the multivariate logistic regression analysis. The β coefficients values were adjusted to the nearest integer to develop a simple integer-based modeling scale. ROC curves were constructed to determine the diagnostic ability of the scale. The optimal cutoff score of the scale was calculated using the Youden’s index.

For statistical analysis, Statistical Package for the Social Sciences was used (version 25.0, IBM Corp., Armonk, NY, USA). Statistical significance was defined as a probability value of *p* < 0.05.

## 3. Results

### 3.1. Participants’ Characteristics 

The average age of the 161 subjects was 69.88 ± 7.23 years (mean ± SD). Of the subjects, 90 (55.9%) were women and 71 (44.1%) were men; also, the subjects consisted of 70 *APOE*-ε4 carriers (43.5%). A total of 78 participants (48.4%) showed Aβ positivity in amyloid PET.

Table 1 shows the demographic findings of the subjects according to the amyloid status. There was a significant difference in the age (*p* = 0.034), but not in sex (*p* = 0.086) and education (*p* = 0.685) between the Aβ+ and Aβ− groups. However, there were no significant differences in the cardiovascular risk factors, such as hypertension, diabetes mellitus, hyperlipidemia, and heart disease, as well as stroke and cancer.

Table 2 demonstrates the clinical characteristics such as the cognition status, MTA aVRS, and *APOE* genotypes of the subjects according to the amyloid status. Among neuropsychological tests, Aβ+ subjects showed significantly lower z-scores of SVLT-DR, RCFT-DR, and K-BNT than Aβ− subjects (SVLT-DR: *p* < 0.001; RCFT-DR: *p* < 0.001; K-BNT: *p* = 0.029). Compared to the Aβ− group, the Aβ+ group was more likely to have both verbal and visual memory impairments rather than either visual or verbal memory alone (*p* < 0.001). The K-MMSE score of the Aβ+ group was lower than that of the Aβ− group (*p* < 0.001). Aβ+ subjects had higher global CDR and CDR-SB scores than Aβ− subjects (*p* = 0.002; *p* < 0.001). Aβ+ subjects had lower MTA aVRS than Aβ− subjects (*p* < 0.001). The proportion of *APOE*-ε4 carriers among Aβ+ subjects was significantly higher than that among Aβ− subjects (*p* < 0.001).

### 3.2. Development of a New Predictive Scale for Amyloid PET Positivity 

Potential predictors that showed significant differences between the Aβ+ and Aβ−groups were extracted, including SVLT-DR, RCFT-DR, K-BNT, K-MMSE, global CDR, CDR-SB, MTA aVRS, *APOE* genotype, and age. Correlation analysis of the variables demonstrated that K-MMSE had a correlation with SVLT-DR (*p* < 0.001), RCFT-DR (*p* < 0.001), K-BNT (*p* = 0.030), global CDR (*p* < 0.001), and CDR-SB (*p* < 0.001). Global CDR and CDR-SB also showed significant correlation with SVLT-DR, RCFT-DR, K-BNT, and K-MMSE. Thus, K-MMSE, global CDR, and CDR-SB were excluded when constructing the models.

Table 3 shows the results of univariate logistic regression analyses to assess the association between each potential predictor and amyloid PET positivity after adjusting for age. SVLT-DR (adjusted odds ratio (aOR) 3.237, 95% CI 1.407–7.448), RCFT-DR (aOR 2.997, 95% CI 1.414–6.351), MTA aVRS (aOR 3.301, 95% CI 1.611–6.766), *APOE*-ε4 carriers (aOR 12.253, 95% CI 5.410–27.753), and age (odds ratio (OR) 2.340, 95% CI 1.207–4.538) were associated with amyloid PET positivity. However, K-BNT (*p* = 0.207) did not demonstrate significance in the univariate analysis. Since all aMCI patients subjects already had either impaired visual or verbal memory, the aOR of both aMCI with both visual and verbal memory impairment was calculated and compared to single aMCI with only visual or verbal memory impairment (aOR 7.579, 95% CI 3.754–15.299).

Both aMCI with both visual and verbal memory impairment, MTA aVRS, and *APOE* genotype were included in the multivariate analyses after adjustment for age. These predictors were combined to develop the following models:

Model 1: included age, both aMCI.
Model 2: included age, both aMCI, MTA aVRS.
Model 3: included age, both aMCI, *APOE* genotype.
Model 4: included age, both aMCI, MTA aVRS, *APOE* genotype.

Multivariate analyses showed that predictors in all models had significant ORs (Table 4).

**Figure 1:**
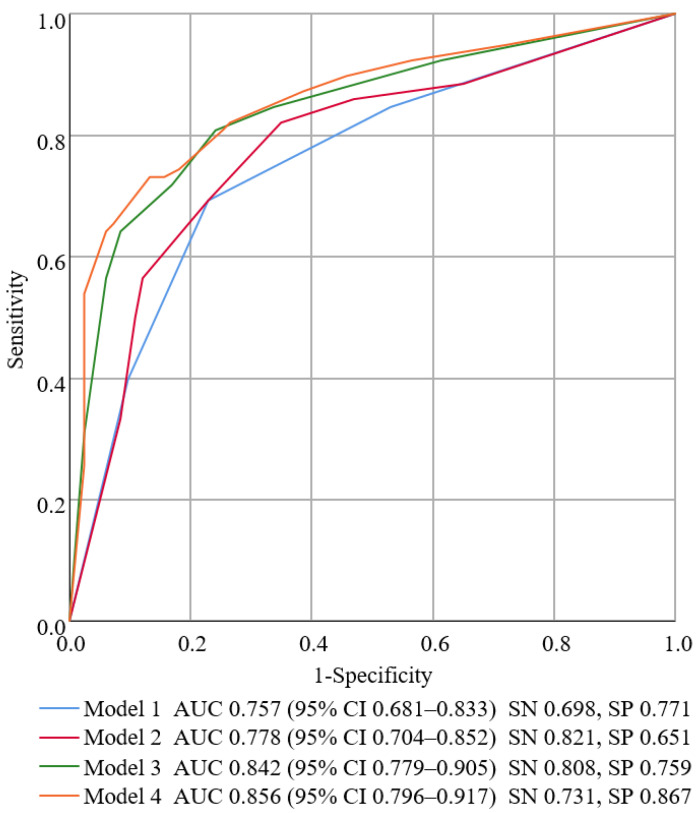
Receiver operating characteristic (ROC) curves of the models to predict amyloid positron emission tomography (PET) positivity. The ROC curves and the corresponding area under the curve (AUC) with 95% confidence interval (CI), sensitivity (SN), and specificity (SP) are shown for all the models. Among the four models, model 4 demonstrates the highest AUC value of 0.856, along with a sensitivity of 73.1% and specificity of 86.7%.

Figure 1 shows the ROC curves and AUC values of the four models. Among the models, model 4 had the highest AUC value (0.856, 95% CI 0.796–0.917) and could be selected as the most appropriate model for predicting amyloid PET positivity (sensitivity: 73.1%, specificity: 86.7%). Model 4 included the following predictors: (1) age older than 70 years (aOR 1.443, 95% CI 0.600–3.473); (2) both aMCI compared to single aMCI (aOR 3.485, 95% CI 1.530–7.940); (3) MTA aVRS value greater than or equal to 2 (aOR 2.668, 95% CI 1.106–6.436); and (5) *APOE*-ε4 carrier (aOR 9.254, 95% CI 3.971–21.566). VIF values of all variables were <4 (age: 1.293; both aMCI: 1.305; MTA aVRS: 1.381; *APOE* genotype: 1.241), indicating that there was no multicollinearity among the predictors. The Hosmer−Lemeshow test for model 4 verified that the model fitted well with the data (χ^2^ = 4.787, *p*-value = 0.686).

To develop a new predictive scale based on a simple integer scoring system, the regression β coefficients generated from the multivariable analysis of model 4 were simplified. The β coefficients were divided by the β coefficient of MTA aVRS, which was the lowest at 0.981 among all predictors. They were rounded to the nearest integer for the scoring system. Table 5 demonstrates the new predictive scale, configured to score 1 point each for age 70 or older, both aMCI compared to single aMCI, MTA aVRS above 2, and 2 points for *APOE*-ε4 carrier, based on the value of the β coefficients. In the scale, a higher score had a higher probability of Aβ positivity (score range: 0–5).

**Figure 2:**
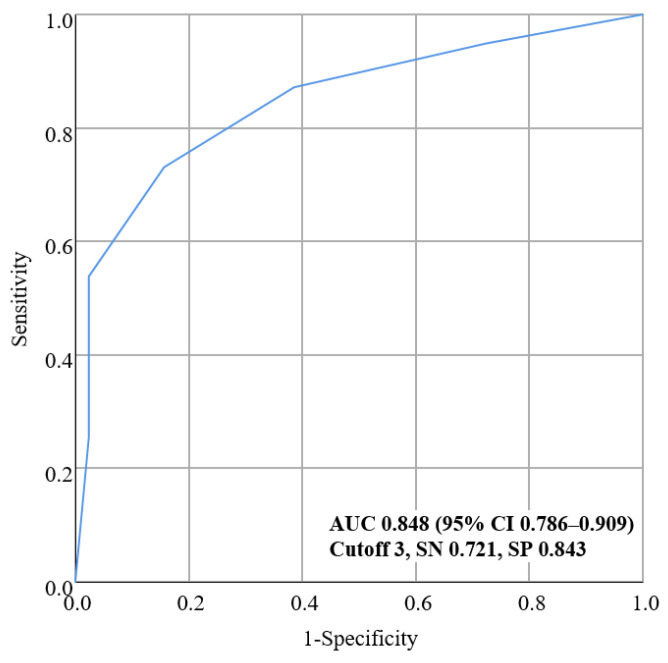
Receiver operating characteristic (ROC) curve of the new predictive scale for amyloid PET positivity. The ROC curve and corresponding area under the curve (AUC) with 95% confidence interval (CI), sensitivity (SN), and specificity (SP) are shown. The AUC value of the scale was high at 0.847, and the sensitivity and specificity were also high at 72.1% and 84.3%, respectively.

ROC curve analysis (Figure 2) shows the performance of the β coefficient-based scoring modeling score to differentiate Aβ+ from Aβ− with an AUC value of 0.848 (95% CI 0.786–0.909). The optimal cutoff point of the modeling score was ≥3 according to the highest Youden’s index with a sensitivity of 72.1% and specificity of 84.3%.

### 3.3. Validation Analysis

**Figure 3:**
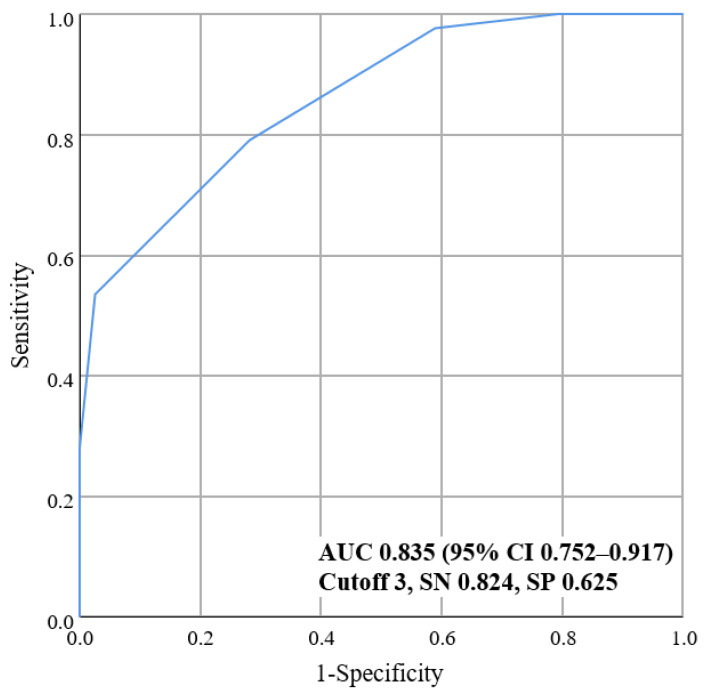
Receiver operating characteristic (ROC) curve of the new predictive scale in the validation cohort. The ROC curve and the corresponding area under the curve (AUC) with 95% confidence interval (CI), sensitivity (SN), and specificity (SP) are shown. In the validation cohort, the AUC value was as high as 0.835, and the sensitivity and specificity were 82.4% and 62.5%, respectively.

Among the 98 aMCI patients subjects in the validation cohort, 34 (34.7%) were amyloid PET positive. Figure 3 shows the prediction performance of the newly developed scale in the validation cohort with an AUC value of 0.835 (95% CI 0.752–0.917). At the optimal cutoff point of the scale, which was ≥3, sensitivity was 82.4% and specificity was 62.5%.

## 4. Discussion

Our study developed a new predictive scale for detecting amyloid PET positivity with easily accessible variables in aMCI subjects. To our knowledge, this is the first predictive scale consisting of integers to identify Aβ+ aMCI subjects by using a combination of the neuropsychological memory domain, *APOE* genotype, and the neurodegenerative marker of MTA VRS. As the prevalence of MCI due to AD increases, this practical scale will be valuable for recognizing Aβ positivity in the prodromal stage.

This predictive scale has the following distinctive strong points. Firstly, the scale can be applied quickly and cost-effectively in clinical practice where amyloid PET evaluation is difficult to access as amyloid PET is a costly and uncommonly used equipment. Several studies have attempted to predict amyloid PET positivity in aMCI participants. These studies required advanced testing tools such as MRI volumetry [14,15,36,37,38] and plasma-based markers [17,39,40], and they have not yet been implemented in clinical trials. However, the predictive scale in the present study comprises readily obtainable variables and is composed of integers. With this new scale, Aβ positivity among aMCI subjects can be predicted easily in clinical settings.

Few studies have attempted to detect Aβ positivity among aMCI subjects with commonly available variables. Kim et al. developed a nomogram for predicting Aβ+ aMCI [41]. This nomogram helps precision medicine at the individual level. However, on the other hand, our predictive scale has the advantage of less variation because each variable is scored as an integer such as 1 or 2 points, regardless of the change in the research subject. Another Aβ positivity prediction model proposed by Lee et al. requires a logit value for each subject to screen, which is challenging to use intuitively in clinical practice [42]. In addition, MTA VRS was not included in the above two studies, although MTA is a characteristic neurodegenerative biomarker of AD [5]. Pekkala et al. also created a model by combining the *APOE* genotype, cognitive status, and MTA [43]. However, their study analyzed a small number of subjects at 48 and recruited not only aMCI but also non-amnestic MCI (naMCI) subjects. The sensitivity and specificity of the model were not as high as the one in the present study. Hence, the comprehensive predictive scale in our study, consisting of integers, may be more useful and convenient for identifying Aβ positive aMCI patients with a higher statistical power than previous studies.

Secondly, this new scale can predict amyloid PET positivity with high specificity in aMCI patients. In the present study, the specificity was high at 86.7% for the optimal cutoff value of the scale. This study was not intended to screen aMCI patients but to develop a scale for predicting Aβ positivity among those diagnosed as aMCI. It will be helpful not only for implementing clinical care but also in the research field. Studies on AD treatment drugs have not been successful, probably due to the difficulty recruiting Aβ+ aMCI patients. The proportion of Aβ+ patients among MCI patients is known to be only 40–60% [44,45]; the present study also had 47.6% amyloid PET-positive subjects in the entire study group. This predictive scale with high specificity may be helpful in precisely selecting patients with Aβ+ aMCI for future clinical studies.

Third, all subjects underwent amyloid PET scans in this study. There have been several observational longitudinal studies on conversion from MCI to AD dementia, but the subjects were diagnosed clinically with probable AD dementia [10,46,47,48]. The present study differs from other studies since all participants were pathologically checked for Aβ load in the brain by PET. Fourth, since this study was based on multi-centers, the results are high generalizable.

Moreover, the results of this study suggest that all predictors in the scale influenced Aβ accumulation independently of each other. Therefore, it is worth looking into each predictor in this study, which is consistent with the results of previous studies. Above all, the *APOE* ε4 allele was found to be an essential factor for the development of Aβ positivity, in line with the results of previous studies [27,49,50]. The *APOE* genotype variable showed the highest aOR in univariate and multivariate analyses (Table 3 and Table 4). The *APOE* genotype also had the highest regression β coefficient generated from model 4. It had twice the points compared to other variables in the predictive scale (Table 5), suggesting that the *APOE* ε4 allele is the most influential predictor of Aβ positivity.

In this study, memory domain impairment in neuropsychological tests was also found to be related to Aβ positivity, concurrent with previous studies [11,20,51]. SVLT-DR and RCFT-DR z-scores showed significant associations with amyloid PET positivity in univariate analysis (Table 3), suggesting that verbal and visual memory impairment were associated with amyloid PET positivity in patients with aMCI. Impairment of both verbal and visual memory modalities was also significantly more predictive of Aβ positivity than an impairment of either modality among the patients with aMCI, consistent with the results of previous studies [41,47,52,53,54,55]. Thus, verbal and visual memory scores, especially combined visual and verbal impaired features, can be considered major predictors of Aβ positivity.

Lastly, MTA acted as a useful predictor in this study. MTA can be found not only in normal aging [56], but also in other neurodegenerative diseases such as frontotemporal dementia, hippocampal sclerosis, Lewy body-related pathology, and tau protein aggregation, including argyrophilic grain disease [57,58,59]. There has been a controversy regarding whether the MTA can predict AD. Ten Kate et al. reported that MTA measured by quantitative MRI had a limited added value in detecting Aβ pathology in MCI groups [15]. However, as their study included aMCI and naMCI subjects, the result could be different from those of other studies. In contrast, the present study showed that MTA was significantly associated with Aβ+ aMCI (Table 3 and Table 4). Although another study suggested no significant difference in hippocampal volume atrophy between Alzheimer’s disease and behavioral variant frontotemporal dementia [59], our results showed that MTA could be an essential predictor of Aβ positivity in aMCI patients, if the clinical phenotypes such as age, memory impairment, and the presence of *APOE* ε4 allele were combined for prediction. Our results suggest that MTA as a neuronal injury biomarker of AD [5] can help to recognize amyloid PET positivity in aMCI subjects, consistent with those of previous studies [23,60,61].

This study used a visual scale rather than quantitative analysis regarding MTA measurement. VRS is known to be as accurate as quantitative analysis, and the intra-rater reliability is high [24,43,62,63]. In addition, the aVRS proposed by Kim et al. was applied because it has higher intra-rater reliability than coronal VRS and axial MR images are easier to obtain in clinical settings [30]. Thus, the VRS based on axial MR images was used in this study as it is readily applicable in clinical practice.

Our study has the following limitations. First, when this predictive scale was validated, the specificity was not very high at 62.5%. It may be because the positive rate of amyloid PET in the validation cohort was 34.7%, which is less than the commonly known 40–60% among aMCI patients [44,45]. Nevertheless, the AUC was high at 0.835, indicating the high predictive ability of this scale. Second, since this scale used the SNSB variable, it applies only when SNSB is performed. Further research is required to assess if this scale can be applied by using the z-score of other neuropsychological tests’ verbal and visual memory tests. Third, subjects underwent MRI and PET scans with different equipment, reducing the consistency of results. The MTA aVRS value could have been measured differently, depending on whether it was 1.5-T MRI or 3.0-T MRI. Nevertheless, the fact that the six centers used a variety of machines may be helpful to further generalize the results of this study. Fourth, amyloid positive and negative results of [^18^F] FBB PET were evaluated by visual interpretation, not by standardized uptake value-based quantification. However, the diagnostic accuracy of BAPL and SUVR in [^18^F] FBB PET was relatively consistent in previous studies [64,65]. A nuclear medicine specialist evaluated all [^18^F] FBB PET images at each center. For these reasons, the accuracy of [^18^F] FBB PET results was expected to be high. Furthermore, our additional analysis showed that the BAPL score was correlated with the scores of the new predictive scale (Pearson’s correlation analysis: *r* = 0.678, *p* < 0.001). Further study of the associations between the BAPL score and the scale score can be investigated. Finally, although memory impairment in MCI is the most distinguishing feature of Aβ positivity [20,66], Aβ positivity in aMCI patients is not necessarily associated with AD. The presence of Aβ positivity reflects only Alzheimer’s pathologic change, and the possibility of other comorbidities such as cerebrovascular disease, frontotemporal lobar degeneration, or α-synucleinopathies cannot be excluded [67].

Despite these limitations, the advantages of this study are as follows: (1) both aMCI, MTA aVRS, and *APOE* genotype were revealed as the critical predictors of Aβ positivity in subjects with aMCI, and (2) the new predictive scale comprising these factors can be easily applied in clinical practice.

## 5. Conclusions

This study shows that verbal and visual memory impairment, MTA, and the *APOE* ε4 allele are associated with Aβ load in the brain of aMCI patients. Integration of these predictors was used to develop a new predictive scale of integers to detect Aβ positivity in aMCI patients with a cutoff score of ≥3. The scale can be used conveniently to predict AD and prognosis of aMCI patients in clinical practice.

## Figures and Tables

**Table 1:** Participants’ demographic findings according to the amyloid status.

	Total (*n* = 161)	Aβ− (*n* = 83)	Aβ+ (*n* = 78)	*p*-Value
Age (years)	69.88 ± 7.23	68.71 ± 7.57	71.12 ± 6.67	0.034
Sex (men:women)	71:90	42:41	29:49	0.086
Education (years)	11.09 ± 4.36	11.23 ± 4.38	10.95 ± 4.36	0.685
Family history of dementia	48/153 (31.4%)	27/81 (33.3%)	21/72 (29.2%)	0.579
Hypertension	74/152 (49.0%)	40/76 (53.3%)	34/76 (44.7%)	0.291
Diabetes mellitus	33/152 (21.9%)	20/76 (26.7%)	13/76 (17.1%)	0.155
Hyperlipidemia	63/152 (41.4%)	34/76 (44.7%)	29/76 (38.2%)	0.410
Heart disease	4/151 (2.6%)	3/75 (4.0%)	1/76 (1.3%)	0.367
Stroke	2/151 (1.3%)	2/75 (2.7%)	0/76 (0.0%)	0.245
Cancer	13/137 (9.5%)	3/65 (4.6%)	10/72 (13.9%)	0.064

Abbreviations: Aβ, amyloid-β; Aβ−, amyloid PET negative, Aβ+, amyloid PET positive.

**Table 2:** Participants’ cognition, MTA, and *APOE*-ε4 according to the amyloid status.

	Total (*n* = 161)	Aβ− (*n* = 83)	Aβ+ (*n* = 78)	*p*-Value
SVLT-DR z-score	−1.51 ± 1.06	−1.18 ± 0.96	−1.86 ± 1.06	<0.001
RCFT-DR z-score	−1.19 ± 1.02	−0.78 ± 1.03	−1.63 ± 0.82	<0.001
DST-forward z-score	0.03 ± 1.05	0.17 ± 1.12	−0.12 ± 0.96	0.090
K-BNT z-score	−0.39 ± 1.22	−0.18 ± 1.27	−0.12 ± 0.96	0.029
RCFT-copy	−0.65 ± 1.65	−0.58 ± 1.35	−0.72 ± 1.93	0.576
COWAT-phonemic z-score	−0.29 ± 0.82	−0.23 ± 0.84	−0.35 ± 0.79	0.341
K-CWST-CR z-score	−0.41 ± 1.11	−0.33 ± 1.01	−0.49 ± 1.22	0.366
DSC z-score	0.09 ± 0.98	0.10 ± 1.01	0.08 ± 0.96	0.930
K-TMT-B z-score	−0.63 ± 1.84	−0.54 ± 1.78	−0.75 ± 1.92	0.493
Visual aMCI	38 (23.6%)	28 (33.7%)	10 (12.8%)	<0.001
Verbal aMCI	50 (31.1%)	36 (43.4%)	14 (17.9%)	
Both aMCI	73 (45.3%)	19 (22.9%)	54 (69.2%)	
K-MMSE	25.80 ± 2.79	26.88 ± 2.20	24.64 ± 2.90	<0.001
Global CDR	0.42 ± 0.25	0.36 ± 0.24	0.48 ± 0.25	0.002
CDR-SB	1.76 ± 1.50	1.31 ± 1.24	2.22 ± 1.61	<0.001
MTA aVRS ≥ 2	81 (50.3%)	29 (34.9%)	52 (66.7%)	<0.001
*APOE*-ε4 carrier (%)	70 (43.5%)	14 (16.9%)	56 (71.8%)	<0.001

Abbreviations: Aβ, amyloid-β; Aβ−, amyloid PET negative, Aβ+, amyloid PET positive; SVLT-DR, Seoul Verbal Learning Test-delayed recall; RCFT-DR, Rey Complex Figure Test-delayed recall; DST-forward, Digit Span Test-forward; K-BNT, Korean-Boston Naming Test; COWAT-phonemic, Controlled Oral Word Association Test-phonemic; K-CWST-CR, Korean-Color Word Stroop Test-color reading; DSC, Digit Symbol Coding; K-TMT-B, Korean-Trail Making Test-part B; aMCI, amnestic mild cognitive impairment; K-MMSE, Korean version-Mini-Mental State Examination; CDR, Clinical Dementia Rating scale; CDR-SB, sum of boxes of CDR; MTA, medial temporal lobe atrophy; aVRS, axial visual rating scale; *APOE*, apolipoprotein E.

**Table 3:** Univariate models to predict for amyloid PET positivity.

Variables	OR (95% CI)	*p*-Value
SVLT-DR (Ref. > −1 SD) *	3.652 (1.637–8.149)	0.002
RCFT-DR (Ref. > −1 SD) *	3.333 (1.616–6.876)	0.001
Both aMCI (Ref. single aMCI)	7.579 (3.754–15.299)	<0.001
K-BNT (Ref. > −1 SD) *	1.684 (0.840–3.377)	0.142
MTA aVRS (Ref. < 2) *	3.724 (1.940–7.149)	<0.001
*APOE*-ε4 carrier (Ref. noncarrier) *	12.545 (5.884–26.749)	<0.001
Age (Ref. < 70)	1.916 (1.022–3.594)	0.043

* Age-adjusted; Abbreviations: OR, odds ratio; CI, confidence interval; SVLT-DR, Seoul Verbal Learning Test-delayed recall; SD, standard deviation; RCFT-DR, Rey Complex Figure Test-delayed recall; aMCI, amnestic mild cognitive impairment; DST-forward, Digit Span Test-forward; K-BNT, Korean-Boston Naming Test; MTA, medial temporal lobe atrophy; aVRS, axial visual rating scale; *APOE*, apolipoprotein E.

**Table 4:** Multivariate analyses of models combining predictors for amyloid PET positivity.

	Model 1 OR (95% CI)	Model 2 OR (95% CI)	Model 3 OR (95% CI)	Model 4 OR (95% CI)
Both aMCI (Ref. single aMCI)	7.368 *** (3.633–14.943)	6.348 *** (3.080–13.083)	4.114 ** (1.850–9.150)	3.485 ** (1.530–7.940)
MTA aVRS (Ref. < 2)	NI	2.533 * (1.177–5.449)	NI	2.668 * (1.106–6.436)
*APOE*-ε4 carrier (Ref. noncarrier)	NI	NI	9.090 *** (3.988–20.722)	9.254 *** (3.971–21.566)
Age (Ref. < 70)	1.684 (0.833–3.405)	1.228 (0.571–2.638)	2.082 (0.933–4.645)	1.443 (0.600–3.473)

* *p* < 0.05, ** *p* < 0.01, *** *p* < 0.001, difference between Aβ+ and Aβ−; Abbreviations: OR, odds ratio; CI, confidence interval; aMCI, amnestic mild cognitive impairment; MTA, medial temporal lobe atrophy; aVRS, axial visual rating scale; *APOE*, apolipoprotein E; NI, not included.

**Table 5:** A new predictive scale for predicting amyloid PET positivity.

Predictors		β Coefficient	Scoring System
Age	<70	0	0
	≥70	0.367	1
Memory impairment	Single (verbal or visual)	0	0
	Both (verbal and visual)	1.249	1
MTA aVRS	<2	0	0
	≥2	0.981	1
*APOE*-ε4 allele	Noncarrier	0	0
	Carrier	2.225	2
Total			0~5

Abbreviations: SVLT-DR, Seoul Verbal Learning Test-delayed recall; RCFT-DR, Rey Complex Figure Test-delayed recall; MTA, medial temporal lobe atrophy; aVRS, axial visual rating scale; *APOE*, apolipoprotein E.

## Data Availability

The raw data supporting the conclusion of this article will be made available by the authors without undue reservation.
